# Kinetics and dissolution of intratracheally administered nickel oxide nanomaterials in rats

**DOI:** 10.1186/s12989-017-0229-x

**Published:** 2017-11-28

**Authors:** Naohide Shinohara, Guihua Zhang, Yutaka Oshima, Toshio Kobayashi, Nobuya Imatanaka, Makoto Nakai, Takeshi Sasaki, Kenji Kawaguchi, Masashi Gamo

**Affiliations:** 1 National Institute of Advanced Industrial Science and Technology (AIST), Tsukuba, Ibaraki 305-8569 Japan; 2 Chemicals Evaluation and Research Institute (CERI), Hita, Oita 877-0061 Japan; 3 Chemicals Evaluation and Research Institute (CERI), Bunkyo, Tokyo, 112-0004 Japan; 4 National Institute of Advanced Industrial Science and Technology (AIST), Tsukuba, Ibaraki 305-8565 Japan

**Keywords:** Toxicokinetics, Intratracheal administration, Clearance rate constant, Lymph node, Dissolubility, Artificial biological fluid (Gamble’s solution)

## Abstract

**Background:**

The toxicokinetics of nanomaterials are an important factor in toxicity, which may be affected by slow clearance and/or distribution in the body.

**Methods:**

Four types of nickel oxide (NiO) nanoparticles were single-administered intratracheally to male F344 rats at three doses of 0.67–6.0 mg/kg body weight. The rats were sacrificed under anesthesia and the lung, thoracic lymph nodes, bronchoalveolar lavage fluid, liver, and other organs were sampled for Ni burden measurement 3, 28, and 91 days post-administration; Ni excretion was measured 6 and 24 h after administration. Solubility of NiO nanoparticles was determined using artificial lysosomal fluid, artificial interstitial fluid, hydrogen peroxide solution, pure water, and saline. In addition, macrophage migration to trachea and phagosome-lysosome-fusion rate constants were estimated using pulmonary clearance and dissolution rate constants.

**Results:**

The wire-like NiO nanoparticles were 100% dissolved by 24 h when mixed with artificial lysosomal fluid (dissolution rate coefficient: 0.18/h); spherical NiO nanoparticles were 12% and 35% dissolved after 216 h when mixed with artificial lysosomal fluid (1.4 × 10^−3^ and 4.9 × 10^−3^/h). The largest irregular-shaped NiO nanoparticles hardly dissolved in any solution, including artificial lysosomal fluid (7.8 × 10^−5^/h). Pulmonary clearance rate constants, estimated using a one-compartment model, were much higher for the NiO nanoparticles with a wire-shape (0.069–0.078/day) than for the spherical and irregular-shaped NiO nanoparticles (0–0.012/day). Pulmonary clearance rate constants of the largest irregular-shaped NiO nanoparticles showed an inverse correlation with dose. Translocation of NiO from the lungs to the thoracic lymph nodes increased in a time- and dose-dependent manner for three spherical and irregular-shaped NiO nanoparticles, but not for the wire-like NiO nanoparticles. Thirty-five percent of the wire-like NiO nanoparticles were excreted in the first 24 h after administration; excretion was 0.33–3.6% in that time frame for the spherical and irregular-shaped NiO nanoparticles.

**Conclusion:**

These findings suggest that nanomaterial solubility differences can result in variations in their pulmonary clearance. Nanoparticles with moderate lysosomal solubility may induce persistent pulmonary inflammation.

**Electronic supplementary material:**

The online version of this article (10.1186/s12989-017-0229-x) contains supplementary material, which is available to authorized users.

## Background

There are growing concerns about the toxicity of nanomaterials owing to the lack of information on their potential risks in workers and the general population. Nanomaterials with the same chemical formula may exert different toxicities, depending on physicochemical characteristics such as size, shape, and crystalline structure. Studies have compared the toxicities of nanomaterials with different physicochemical properties [1]. Pulmonary clearance and translocation to extrapulmonary organs offer valuable insights into the inhalation toxicity of nanomaterials. Recently, we showed that six types of TiO_2_ nanoparticles of different sizes and shapes had similar pulmonary clearance rate constants, while TiO_2_ nanoparticles with an Al(OH)_3_ coating had much lower pulmonary clearance rate constants [2] and displayed higher toxicity [3]. TiO_2_ nanoparticle toxicity increases with decreasing particle size [3]. Extensive inflammation was observed in rats intratracheally instilled with nickel oxide (NiO) nanoparticles, but was minimal in rats intratracheally instilled with NiO submicron-sized particles [4, 5].

Although NiO is often considered a poorly soluble Ni compound, some NiO can be dissolved [6]. Inhaled soluble Ni compounds are rapidly cleared from the lung and exhibit higher toxicity, while inhaled insoluble NiO is slowly cleared from the lungs and exhibits lower toxicity [7–10]. Ni-containing compounds can induce pulmonary inflammation [11–13], which could be related to the dissolution rate [13]. Solubility and cytotoxicity are higher for black NiO than for green NiO, even though the particle size is identical [14, 15]. Therefore, the toxicity and pulmonary clearance of NiO nanoparticles could be associated with particle size and solubility. In addition, in vivo nanoparticle dissolution should be considered, as well as water solubility.

This study evaluated the relationship between the toxicokinetics and biological solubility of NiO using an artificial biological fluid, as biological solubility is difficult to determine in situ. Further, we measured pulmonary clearance kinetics, extrapulmonary translocation, and excretion of four NiO nanomaterials, which differed in particle size and biological solubility, after intratracheal administration in rats. Finally, the pulmonary clearance and lung-to-lymph translocation rate constants of these four nanomaterials were compared for a range of doses.

## Methods

### Preparation and characterization of NiO suspensions

Four NiO particles (A, B, C, and D) were used in the present study. The names and manufacturers of these NiO particles, as well as the suspension characteristics, including primary particle size, surface area, and size of the agglomerates in suspension, are shown in Table 1.

**Table 1:** Characteristics of the NiO particles used in the study

	Name (Manufacturer)	Crystalline	Shape	Primary particle size^a^ [nm]	Specific surface area^b^ [m^2^/g]	Converted spherical primary particle size based on the specific surface area [nm]	Number-based agglomerate particle size^c^ (DLS measurement) [nm]	SEM/TEM picture
A	US3352 (US Research Nanomaterials, Inc., TX, USA)	NaCl type	Spherical	20 ± 8	51	18	49	
B	NovaWireNiO1 (Novarials Co., MA, USA)	NaCl type	Wire	Length 240 Diameter 29	180	5.0	Impossible determination^e^	
C	I small particle (Kusaka Rare Metal Products Co., LTD., Tokyo, Japan)	NaCl type	Irregular	140 ± 67	6.6	140	1600	
D	Ni(II) Oxide Nanopowder (Sigma-Aldrich Co. LLC., MO, USA)	NaCl type	Spherical	Impossible observation^d^	93	9.6	39	

^a^Determined by SEM (scanning electron microscopy, S4800, Hitachi High-Technologies Co., Tokyo, Japan) or TEM (transmission electron microscopy, JEM-2010, JEOL, Tokyo, Japan) of 500 particles for each material

^b^Determined by Brunauer-Emmett-Teller (BET) surface area analysis (GEMINI VII, Shimadzu Co., Kyoto, Japan) after drying

^c^Determined by dynamic light scattering (DLS) (Zetasizer nano-ZS; Malvern Instruments Ltd., Worcestershire, UK)

^d^ The small NiO D particle size, caused the particles to aggregate when the suspension was dried. Consequently, particle dimensions were difficult to ascertain even with suspension dilution and spraying

^e^ Reproducibiliy of dynamic light scattering (DLS) measurement for NiO B was not feasible

NiO particles (2 g) were sonicated in 50 mL pure water for 2 h, followed by centrifugation at 1000 *g* for 30 min at 20°C. The supernatant was collected as a stock suspension to remove the large aggregate of particles. Suspensions of 0.67, 2.0, and 6.0 mg/mL for animal tests and 2.0 mg/mL for biological solubility tests were prepared by diluting the stock suspension with pure water. The concentration of the stock suspension was determined by a standard weight analysis, where the weight-loss of the suspension was measured using a balance (AUW220D; Shimadzu Co., Kyoto, Japan) after drying at 200 °C in a thermostatic chamber (ON-300S; Asone Co., Osaka, Japan).

### Biological solubility of NiO nanoparticles

For these experiments, six solutions were used as artificial biological fluids: Saline; hydrogen peroxide (266.7 and 13.3 μmol/L); artificial interstitium solution (Gamble’s solution); artificial lysosomal solution; pure water. The final concentrations of the hydrogen peroxide solution (200 and 10 μmol/L) were selected based on previous studies [16–18]. The artificial interstitium and artificial lysosomal fluids were prepared using the method shown in Additional file 1.

The nanoparticle suspension (10 mL of 2.0 mg/mL) and artificial biological fluids (30 mL) were mixed and shaken at 200 rpm. At 0.5–216 h after the mixing period (12 time points), 2.5 mL fluid were sampled and filtered at 6000 *g* for 30 min with an ultrafilter (50,000 molecular weight cut off [MWCO]; VS2032, Sartorius Stedim Biotech, Aubagne, France) to separate the dissolved Ni ions; a molecular weight of 50,000 is equivalent to 6–7 nm. Ultrapure water (1 mL) was then added to the ultrafiltrate, and the samples were centrifuged at 6000 *g* for 15 min; these steps were performed twice. The Ni ion concentration (ng/mL) was then analyzed, fitted to the curve, and the solution equilibrium was expressed using the following equation:

1 $$ \mathrm{Solid}\;\underset{k'}{\overset{k_{\mathrm{dis}}}{\rightleftarrows }}\;\mathrm{ion} $$

The dissolution and solidification rate coefficients, *k*_*dis*_ (/h) and *k’* (/h), respectively, were estimated using the least-squares approach with the solver function in Excel 2007.

### Experimental procedure for animal testing

Two hundred and forty male F344/DuCrlCrlj rats (12 weeks old; mean body weight 256 ± 11 g) obtained from Charles River Laboratories Japan, Inc. (Kanagawa, Japan) were anesthetized by isoflurane inhalation and intratracheally administered pure water (as the negative control) or NiO suspensions (0.67, 2.0, or 6.0 mg/kg body weight [BW]) at 1 mL/kg BW using a MicroSprayer® Aerosolizer (model IA-1B-R for Rat; Penn-Century, Inc., Wyndmoor, PA, USA). Five rats in each group were euthanized by exsanguination from the abdominal aorta under intraperitoneal pentobarbital anesthesia (50 mg/kg BW) on days 3, 28, and 91 post-NiO particle administration. Thereafter, the lungs were lavaged twice with 7 mL physiological saline, as previously described [2]. After the bronchoalveolar lavage fluid (BALF) sampling, the trachea, lungs, right and left posterior mediastinal lymph nodes, parathymic lymph nodes, liver, kidneys, spleen, and brain of each animal were dissected, rinsed with saline, and weighed. The observation period of 91 days was chosen because the fast clearance pathway could become less attributed to pulmonary clearance more than 91 days after administration. The effect of slow clearance can be evaluated in 91 days according to previous studies that reported the half time of fast clearance as 25–27 days [19] and 23–50 days [20], and half time of slow clearance as 224 days [19] and 75–845 days [20]. They found the differences in fast clearance rates, between doses and mammalian species, to be small.

Additional tests were conducted using metabolism cages to evaluate excretion in five rats from each group administered NiO particles (2.0 mg/kg BW). The feces and urine were collected between 0 and 6 h and 6–24 h post-NiO administration. The rats were euthanized and dissected 6 h and 24 h post-NiO administration. In addition to the organs listed above, the small intestine, large intestine, esophagus, and stomach were dissected after collection of BALF and gastrointestinal contents. Extracellular Ni ion fractions ([extracellular Ni ions]/[extracellular Ni particles + extracellular Ni ions + intracellular Ni particles + intracellular Ni ions]) in BALF were determined after ultrafiltration.

All animals were treated in accordance with the guidelines for animal experiments in our laboratory, which adhere to the guidelines of the Ministry of the Environment, Ministry of Health, Labour and Welfare, Ministry of Agriculture, Forestry and Fisheries, and Ministry of Education, Culture, Sports, Science and Technology. The experiments were approved by the Animal Care and Use Committee, Chemicals Evaluation and Research Institute, and the Institutional Animal Care and Use Committee of the National Institute of Advanced Industrial Science and Technology.

### Pulmonary NiO clearance and translocation rate coefficients

**Fig. 1:**
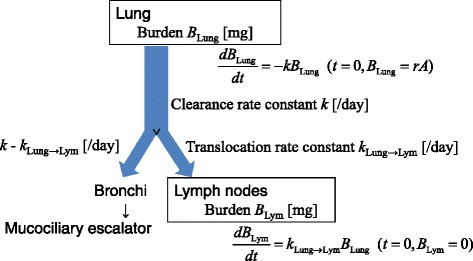
One-compartment model for the clearance of NiO nano and submicron particles. This model is expressed using a first-order decay equation with rate constant *k*

We used the previously described [2] model shown in Fig. 1 to express the pulmonary clearance and translocation to thoracic lymph nodes. The rate constants (/day) for pulmonary clearance (*k*_Lung_) and translocation from the lung to thoracic lymph node (*k*_Lung → Lym_) were estimated using eqs. (2) and (3), according to Shinohara et al. [2]:

2 $$ {\displaystyle \begin{array}{l}\frac{dB_{\mathrm{Lung}}}{dt}=-{k}_{\mathrm{Lung}}{B}_{\mathrm{Lung}}\\ {}\kern1.5em \left(t=0:{B}_{\mathrm{Lung}}= rD\right)\ \end{array}} $$

3 $$ {\displaystyle \begin{array}{l}\frac{dB_{\mathrm{Lym}}}{dt}={k}_{\mathrm{Lung}\to \mathrm{Lym}}{B}_{\mathrm{Lung}}\\ {}\kern1.5em \left(t=0:{B}_{\mathrm{Lym}}=0\right)\ \end{array}} $$

where *B*_Lung_ and *B*_Lym_ were the lung and total thoracic lymph node burdens of NiO, respectively; *D* was the administered dose of NiO (μg); *t* was the time elapsed after administration (days), and *r* was the fraction that reached the alveolar region following NiO administration. The least-squares approach was used for curve-fitting.

### Analysis

Samples were homogenized in ultrapure water and then acid-treated prior to determining the Ni content using inductively coupled plasma-mass spectrometry (ICP-MS), under the conditions shown in Table 2. We altered the acid-treatment methods for NiO C and D because the recovery efficiency for NiO C was not high when using the method for NiO A and B.

**Table 2:** Instruments and conditions used for homogenization, acid treatment, and analysis

Homogenate		
Volume of pure water for homogenate	Liver	10 mL
	Other organs	2 mL
	Face	2 mL
	Food	2 mL
	Gastrointestinal contents	2 mL
Electric homogenizer	PT10–35 (Kinematica AG, NS-50, and NS-52; Microtec Co. Ltd., Chiba, Japan)	
Acid treatment for NiO A and B		
Volume of samples and acid	Homogenate sample	1.0 g
	HNO_3_ (68%)	1.0 mL
Ashing	180 °C (2 h) ⇒ 350 °C (2 h) ⇒ 550 °C (3 h)	
Electric furnace	TFF45-C (Tokyo Technological Labo Co. Ltd., Kanagawa, Japan)	
Acid treatment for NiO C and D		
Volume of samples and acid	Homogenate sample	1.0 g
	HNO_3_ (68%)	0.5 mL
	H_2_O_2_	0.2 mL
Heating	180 °C (20 min)	
Microwave sample preparation instrument	ETHOS 1 (Milestone Srl, Sorisole, Italy) or Speedwave 4 (Berghof, Eningen, Germany)	
Analysis		
ICP-MS	ELEMENT 2/ ELEMENT XR (Thermo Fisher Scientific, Waltham, MA, USA)	
RF output	1200 W	
Target mass (m/z)	^60^Ni (mass: 59.9308)	

Good linearity of the calibration curves for Ni using ICP-MS was observed in a 0–20 ng/mL standard solution (R^2^ > 0.999). Recovery efficiencies from Ni standard solution-spiked samples (5 ng/mL) were >90% for most of the samples (Additional file 2). Five nanograms per milliliter of Ni standard solution were added to organ samples every 10–20 samples, and the measured value were corrected by the recovery efficiency. Based on the operation blank for the organ tissue samples, the limit of quantification was 2 ng/g for most organ tissues, 1 ng/mL for BALF, 0.5 ng/mL for blood, and 0.5 ng for trachea and lymph nodes.

### Statistical analysis

Two-way repeated analysis of variance (ANOVA) with Scheffe’s test was used to compare the study group NiO concentrations following an F-test with SPSS 20.0 (IMD SPSS, Armonk, NY, USA). Since the administered doses differed depending on BW, the organ burdens are shown as normalized values where the organ burden was divided by the BW at the time of NiO administration. Percentages of administered doses are also shown for organ burdens.

## Results

### Biological solubility of NiO nanoparticles

**Fig. 2:**
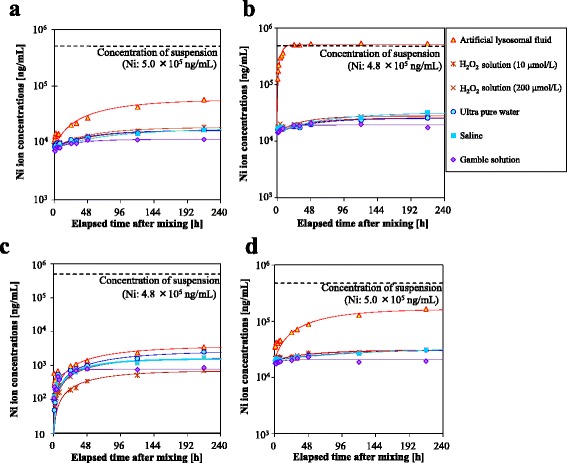
Concentration of dissolved Ni ions in the artificial fluids for four types of NiO. Ultrapure water, saline, hydrogen peroxide (H_2_O_2_) solutions (10 and 200 μM), artificial lysosomal fluid, and Gamble’s solution (artificial interstitium fluid) were used as the artificial fluids. The broken line indicates the Ni concentrations (both particle and ion) in the suspension. **a** NiO A. **b** NiO B. **c** NiO C. **d** NiO D

The Ni ion concentrations increased over time for each NiO nanoparticle tested in each solution (Fig. 2). NiO B was 100% dissolved 24 h after mixing in lysosomal fluid, while only 3.5–6.5% of NiO B had dissolved after 216 h in the other five solutions. In contrast, approximately 11, 0.70, and 33% of NiO A, C, and D, respectively, had dissolved after 216 h in lysosomal fluid, while 2.3–3.7%, 0.14–0.51%, and 3.9–6.3% of NiO A, C, and D, respectively, had dissolved at 216 h in the other five fluids.

The dissolution rate coefficients in lysosomal fluid, *k*_*dis*_, were 2–3 orders of magnitude higher for NiO B than for NiO A, C, and D (Table 3). With the exception of artificial interstitium fluid, the solidification rate coefficients, *k’*, did not differ much between the NiO particles (approximately 1 × 10^−2^/h).

**Table 3:** Dissolution rate coefficient *k* and solidification rate coefficient *k’* of NiO nanoparticles in six solutions

NiO particles	Solutions	*k* (/h)	*k’* (/h)
NiO A	Saline	3.4 × 10^−4^	8.8 × 10^−3^
	Pure water	5.8 × 10^−4^	1.7 × 10^−2^
	H_2_O_2_ (200 μmol/L)	5.0 × 10^−4^	1.4 × 10^−2^
	H_2_O_2_ (10 μmol/L)	5.7 × 10^−4^	1.5 × 10^−2^
	Artificial lysosomal solution	1.4 × 10^−3^	1.1 × 10^−2^
	Artificial interstitium solution (Gamble’s solution)	6.2 × 10^−4^	2.6 × 10^−2^
NiO B	Saline	6.5 × 10^−4^	1.0 × 10^−2^
	Pure water	8.0 × 10^−4^	1.6 × 10^−2^
	H_2_O_2_ (200 μmol/L)	1.2 × 10^−3^	2.3 × 10^−2^
	H_2_O_2_ (10 μmol/L)	9.3 × 10^−3^	1.9 × 10^−2^
	Artificial lysosomal solution	0.18	9.2 × 10^−3^
	Artificial interstitium solution (Gamble’s solution)	3.6 × 10^−3^	9.8 × 10^−2^
NiO C	Saline	5.4 × 10^−5^	1.7 × 10^−2^
	Pure water	5.5 × 10^−5^	1.0 × 10^−2^
	H_2_O_2_ (200 μmol/L)	2.0 × 10^−5^	1.4 × 10^−2^
	H_2_O_2_ (10 μmol/L)	4.7 × 10^−5^	1.4 × 10^−2^
	Artificial lysosomal solution	7.8 × 10^−5^	1.0 × 10^−2^
	Artificial interstitium solution (Gamble’s solution)	1.5 × 10^−4^	9.5 × 10^−2^
NiO D	Saline	3.7 × 10^−4^	4.4 × 10^−3^
	Pure water	5.4 × 10^−4^	7.3 × 10^−3^
	H_2_O_2_ (200 μmol/L)	1.0 × 10^−3^	1.5 × 10^−2^
	H_2_O_2_ (10 μmol/L)	1.1 × 10^−3^	1.5 × 10^−2^
	Artificial lysosomal solution	4.9 × 10^−3^	9.3 × 10^−3^
	Artificial interstitium solution (Gamble’s solution)	4.3 × 10^−3^	9.4 × 10^−2^

### Organ NiO burdens after intratracheal administration

**Fig. 3:**
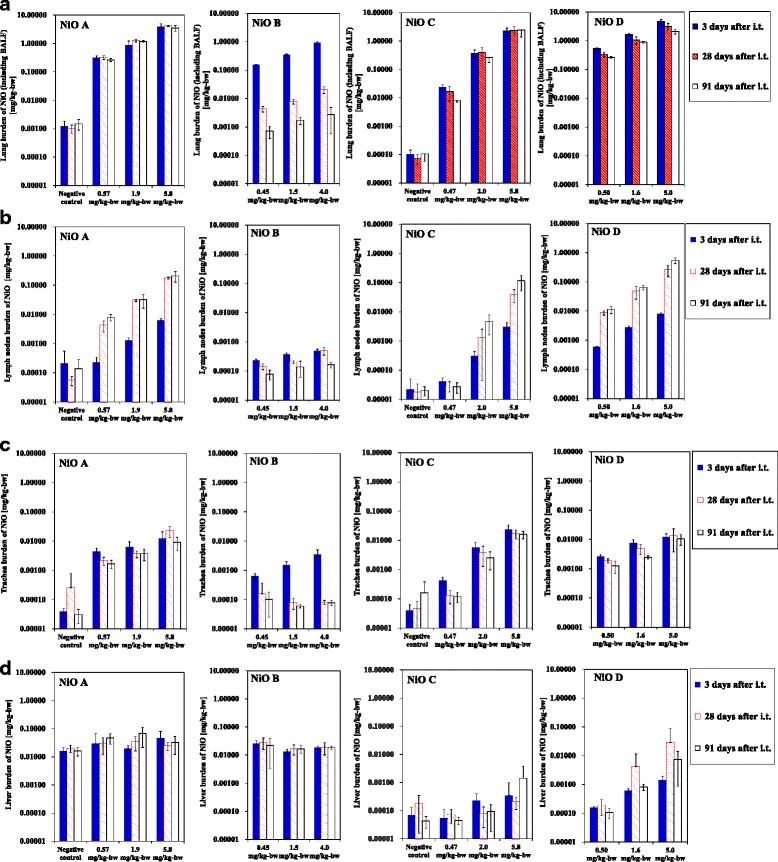
NiO burden per initial body weight at the time of administration in the lung. Bronchoalveolar lavage fluid (BALF) (**a**), total thoracic lymph nodes (right mediastinal lymph node and left and right mediastinal lymph nodes) (**b**), trachea (**c**), and liver (**d**) following intratracheal NiO administration. Horizontal axis indicates the administered dose per initial body weight. The body weights of the rats were 256 ± 11 g. The column and error bars indicate the mean and standard deviation, respectively. Asterisks indicate statistically significant differences, compared with the control group (** *P* < 0.01, * *P* < 0.05). Samples with NiO levels below the quantification limit were assigned values corresponding to half the quantification limit

NiO burdens in BALF and in the lung (after BALF sampling) were significantly higher (*P* < 0.01) in the NiO-treated rats than in the control group from days 3 to 91, except for rats treated with NiO B on day 91 (Fig. 3, Table 4, Additional files 3 and 4). These NiO burdens were dose-dependent. The lung burdens for NiO A, C, and D decreased slowly over time, whereas burdens for NiO B decreased rapidly to <1% of the administered dose by day 28 post-administration. Higher BALF-to-total lung burden ratios were observed at lower doses and with longer observation periods in rats treated with NiO B; this trend was not observed for NiO A, C, or D (Table 5).

**Table 4:** Percentage of NiO burden in each organ per administered dose. Values for the (A) lung, (B) bronchoalveolar lavage fluid (BALF), (C) trachea, and (D) lymph nodes are shown

(A) Lung				
		Percentage of lung NiO burden per administered dose		
		3 days after i.t.	28 days after i.t.	91 days after i.t.
NiO A	0·57 mg/kg-bw	53% ± 11%	56% ± 8.7%	46% ± 5.3%
	1·9 mg/kg-bw	44% ± 21%	66% ± 8.0%	62% ± 4.8%
	5·8 mg/kg-bw	67% ± 17%	71% ± 3.0%	60% ± 14%
NiO B	0·45 mg/kg-bw	33% ± 1.6%	0.87% ± 0.051%	0.28% ± 0.032%
	1·5 mg/kg-bw	22% ± 1.5%	0.48% ± 0.10%	0.12% ± 0.020%
	4·0 mg/kg-bw	22% ± 2.1%	0.50% ± 0.12%	0.088% ± 0.049%
NiO C	0·47 mg/kg-bw	4.8% ± 1.3%	3.4% ± 1.9%	1.7% ± 0.22%
	2·0 mg/kg-bw	18% ± 5.5%	19% ± 9.3%	13% ± 4.5%
	5·8 mg/kg-bw	39% ± 10%	41% ± 14%	42% ± 19%
NiO D	0·50 mg/kg-bw	100% ± 6.3%	64% ± 12%	51% ± 3.8%
	1·6 mg/kg-bw	100% ± 11%	62% ± 16%	54% ± 4.0%
	5·0 mg/kg-bw	90% ± 16%	61% ± 16%	40% ± 7.9%
(B) Bronchoalveolar lavage fluid (BALF)				
		Percentage of BALF NiO burden per administered dose		
		3 days after i.t.	28 days after i.t.	91 days after i.t.
NiO A	0·57 mg/kg-bw	1.2% ± 0.31%	0.51% ± 0.13%	0.59% ± 0.17%
	1·9 mg/kg-bw	0.28% ± 0.17%	0.61% ± 0.068%	0.39% ± 0.034%
	5·8 mg/kg-bw	0.51% ± 0.37%	1.9% ± 0.62%	0.57% ± 0.42%
NiO B	0·45 mg/kg-bw	1.1% ± 0.31%	0.35% ± 0.19%	0.15% ± 0.058%
	1·5 mg/kg-bw	0.94% ± 0.22%	0.11% ± 0.015%	0.058% ± 0.013%
	4·0 mg/kg-bw	0.65% ± 0.19%	0.050% ± 0.015%	0.016% ± 0.0049%
NiO C	0·47 mg/kg-bw	0.078% ± 0.021%	0.11% ± 0.053%	0.088% ± 0.031%
	2·0 mg/kg-bw	0.64% ± 0.81%	0.45% ± 0.20%	0.39% ± 0.092%
	5·8 mg/kg-bw	0.39% ± 0.098%	0.50% ± 0.42%	0.43% ± 0.34%
NiO D	0·50 mg/kg-bw	2.6% ± 0.39%	2.5% ± 1.1%	1.3% ± 0.13%
	1·6 mg/kg-bw	1.8% ± 0.49%	3.9% ± 1.6%	1.8% ± 0.46%
	5·0 mg/kg-bw	1.9% ± 0.63%	1.6% ± 0.74%	1.9% ± 0.54%
(C) Trachea				
		Trachea NiO burden per initial body weight [mg/kg bw]		
		3 days after i.t.	28 days after i.t.	91 days after i.t.
NiO A	0·57 mg/kg-bw	0.77% ± 0.25%	0.38% ± 0.12%	0.30% ± 0.085%
	1·9 mg/kg-bw	0.33% ± 0.17%	0.19% ± 0.050%	0.20% ± 0.085%
	5·8 mg/kg-bw	0.21% ± 0.15%	0.39% ± 0.16%	0.16% ± 0.075%
NiO B	0·45 mg/kg-bw	0.14% ± 0.031%	0.035% ± 0.047%	0.023% ± 0.017%
	1·5 mg/kg-bw	0.10% ± 0.028%	0.0052% ± 0.0021%	0.0040% ± 0.00050%
	4·0 mg/kg-bw	0.086% ± 0.040%	0.0020% ± 0.00033%	0.0015% ± 0.0011%
NiO C	0·47 mg/kg-bw	0.087% ± 0.030%	0.027% ± 0.013%	0.026% ± 0.0097%
	2·0 mg/kg-bw	0.28% ± 0.14%	0.19% ± 0.13%	0.13% ± 0.078%
	5·8 mg/kg-bw	0.40% ± 0.19%	0.29% ± 0.097%	0.28% ± 0.079%
NiO D	0·50 mg/kg-bw	0.52% ± 0.084%	0.38% ± 0.065%	0.25% ± 0.12%
	1·6 mg/kg-bw	0.46% ± 0.16%	0.30% ± 0.12%	0.15% ± 0.017%
	5·0 mg/kg-bw	0.24% ± 0.078%	0.27% ± 0.19%	0.21% ± 0.084%
(D) Lymph nodes				
		Lymph nodes NiO burden per initial body weight [mg/kg bw]		
		3 days after i.t.	28 days after i.t.	91 days after i.t.
NiO A	0·57 mg/kg-bw	0.040% ± 0.019%	0.75% ± 0.30%	1.4% ± 0.32%
	1·9 mg/kg-bw	0.068% ± 0.015%	1.6% ± 0.14%	1.7% ± 0.84%
	5·8 mg/kg-bw	0.10% ± 0.050%	3.0% ± 0.26%	3.6% ± 1.4%
NiO B	0·45 mg/kg-bw	0.051% ± 0.0053%	0.031% ± 0.0069%	0.017% ± 0.0062%
	1·5 mg/kg-bw	0.025% ± 0.0029%	0.014% ± 0.0013%	0.0092% ± 0.0051%
	4·0 mg/kg-bw	0.012% ± 0.0018%	0.012% ± 0.0036%	0.0040% ± 0.00083%
NiO C	0·47 mg/kg-bw	0.0088% ± 0.0026%	0.0062% ± 0.0024%	0.0057% ± 0.0022%
	2·0 mg/kg-bw	0.015% ± 0.0061%	0.065% ± 0.063%	0.23% ± 0.15%
	5·8 mg/kg-bw	0.052% ± 0.023%	0.67% ± 0.31%	2.0% ± 1.1%
NiO D	0·50 mg/kg-bw	0.11% ± 0.0047%	1.7% ± 0.29%	2.2% ± 0.63%
	1·6 mg/kg-bw	0.17% ± 0.020%	3.0% ± 1.4%	4.0% ± 0.69%
	5·0 mg/kg-bw	0.15% ± 0.018%	5.2% ± 2.2%	11% ± 2.4%

**Table 5:** Percentage of Ni in bronchoalveolar lavage fluid (BALF) per Ni in total lung post-administration

		(Ni in BALF) / (Ni in lung and BALF)		
		3 days after i.t.	28 days after i.t.	91 days after i.t.
NiO A	0.57 mg/kg-bw	2.4% ± 1.2%	0.84% ± 0.11%	1.2% ± 0.24%
	1.9 mg/kg-bw	0.59% ± 0.043%	0.89% ± 0.043%	0.60% ± 0.041%
	5.8 mg/kg-bw	0.79% ± 0.52%	2.5% ± 0.70%	0.89% ± 0.48%
NiO B	0.45 mg/kg-bw	3.0% ± 0.88%	31% ± 13%	76% ± 17%
	1.5 mg/kg-bw	4.0% ± 1.0%	19% ± 5.0%	38% ± 2.9%
	4.0 mg/kg-bw	2.8% ± 0.82%	8.9% ± 1.8%	22% ± 5.8%
NiO C	0.47 mg/kg-bw	1.7% ± 0.72%	3.2% ± 1.4%	5.0% ± 1.5%
	2.0 mg/kg-bw	3.2% ± 1.4%	2.5% ± 0.86%	3.1% ± 1.1%
	5.8 mg/kg-bw	5.0% ± 1.5%	1.3% ± 1.4%	1.2% ± 0.93%
NiO D	0.50 mg/kg-bw	2.4% ± 0.29%	3.7% ± 1.7%	2.5% ± 0.33%
	1.6 mg/kg-bw	1.8% ± 0.61%	5.7% ± 1.1%	3.2% ± 0.68%
	5.0 mg/kg-bw	2.0% ± 0.42%	2.7% ± 1.3%	4.8% ± 1.5%

The NiO burdens in most of the thoracic lymph nodes (total burden in the right and left posterior mediastinal lymph nodes and parathymic lymph nodes) were significantly higher in the groups dosed with NiO particles than in the control group (Fig. 3, Table 4, Additional files 3 and 4). Except for NiO B, the NiO burden in the thoracic lymph nodes increased in a dose- and time-dependent manner from day 3 to day 91, with percentage burdens (total burden in the thoracic lymph nodes relative to the administered dose) of 0.015–0.18% (day 3) to 0.12–12% (day 91). In most rats, a higher NiO burden was detected in the right and left mediastinal lymph nodes than in the parathymic lymph node (Additional file 5).

Although the liver NiO burdens in some rats were higher than those in the negative control group, no clear dose- or time-dependency was observed (Fig. 3). No significant differences were observed in the NiO levels of the kidney, spleen, and brain in the NiO-treated and control animals.

### Pulmonary NiO clearance and translocation rate coefficients

**Fig. 4:**
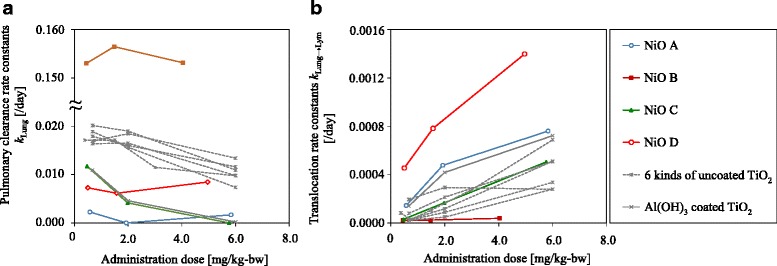
Dose-dependent clearance rate constants (**a**) and lung-to-lymph nodes translocation rate constants (**b**). Broken line in (**a**) shows the 2-month half-life (0.0115/day). The rate constants of seven types of TiO_2_ from a previous study [9] are also described in this figure

The pulmonary NiO clearance rate coefficients, *k*_Lung_, in animals treated with NiO B were much higher than those of animals treated with NiO A, C, or D (Fig. 4 and Table 6). *k*_Lung_ showed an inverse correlation with dose for NiO C, but this relationship was not observed for NiO A, B, or D. The translocation rate coefficients from lung to lymph nodes, *k*_Lung → Lym_, increased in a dose-dependent manner for NiO A, C, and D (Fig. 4 and Table 6). *k*_Lung → Lym_ was highest for NiO D, followed by NiO A, C, and B.

**Table 6:** Pulmonary clearance rate constants and initial fraction of administered NiO that reached the alveolar region

	Dose	Clearance rate constants *k*_Lung_ (1/day)	Half time *t*_1/2_ (day)	Initial fraction reaching the alveolar region *r*	Translocation rate constants *k*_Lung _ _→ Lym_ (1/day)
NiO A	0.57 mg/kg-bw	0.0022	310 day	57%	0.00014
	1.9 mg/kg-bw	0.000	>690 day	58%	0.00048
	5.8 mg/kg-bw	0.0017	410 day	68%	0.00076
NiO B	0.45 mg/kg-bw	0.15	4.5 day	54%	0.000024
	1.5 mg/kg-bw	0.16	4.4 day	38%	0.000024
	4.0 mg/kg-bw	0.15	4.5 day	36%	0.000039
NiO C	0.47 mg/kg-bw	0.012	59 day	5.0%	0.000031
	2.0 mg/kg-bw	0.0041	170 day	20%	0.00017
	5.8 mg/kg-bw	0.000	>690 day	42%	0.00050
NiO D	0.50 mg/kg-bw	0.0073	96 day	97%	0.00045
	1.6 mg/kg-bw	0.0061	110 day	94%	0.00078
	5.0 mg/kg-bw	0.0084	82 day	88%	0.0014

Data estimated from the lung and bronchoalveolar lavage fluid (BALF) burden data, using a one-compartment model

### Evaluation of short-term excretion

**Fig. 5:**
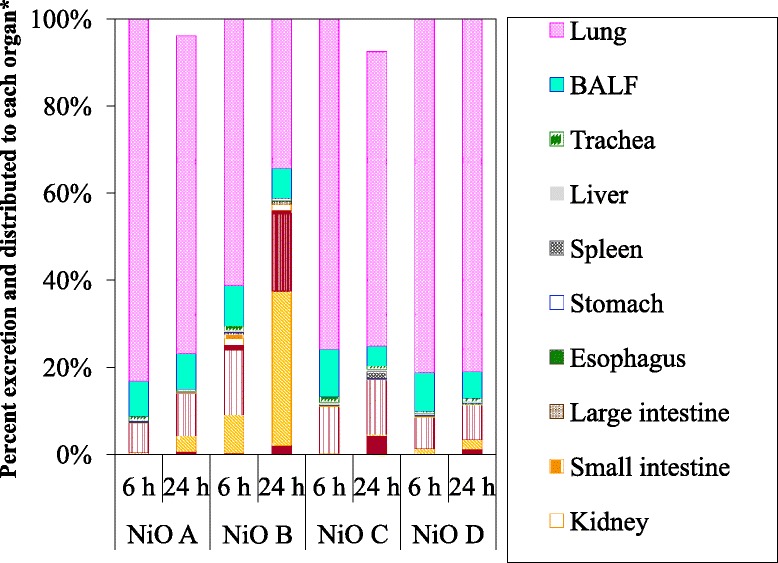
Ni content in organs and excretion 6 h and 24 h post-administration. The total recovery 6 h post-administration was 94, 74, 41, and 90% for NiO A, B, C, and D, respectively

The total organ, feces, urine, and gastrointestinal Ni content 6 h after administration of NiO A, B, C, and D was 96, 74, 44, and 92%, respectively. The distribution and excretion per initial (6-h) total NiO burden are shown in Fig. 5. Ni excretion in urine for up to 24 h was higher for NiO B (35%) than for NiO A (3.6%), while the Ni excretion of NiO D (2.1%) was much higher than that of NiO C (0.33%). Ni excretion levels in the feces for up to 24 h and in the gastrointestinal contents at 24 h were higher for animals treated with NiO B or C than for those exposed to NiO A or D. Although the Ni levels in the esophagus, stomach, small intestine, large intestine, liver, and blood were low (< 1%), the burdens in the kidneys and blood were significantly higher for NiO B than for the other NiO particles. In addition, the extracellular ion fractions in BALF were 8.3 and 9.7% for NiO B at 6 and 24 h after administration, respectively, whereas those for the other three NiO particles were below the detection limit.

### Pulmonary inflammation

**Fig. 6:**
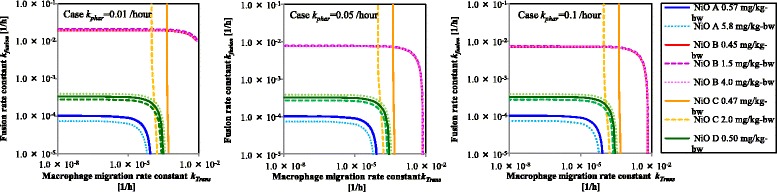
Representative images of H&E-stained lung tissue sections after intratracheal administration of NiO A, B, C, and D (6 mg/kg BW) and vehicle

Representative images of H&E-stained lung tissue sections are shown in Fig. 6. Sustained pulmonary inflammation up to 13 weeks post-administration was observed in NiO A and D, and inflammatory cells (neutrophils and alveolar macrophages) tended to increase over time. For NiO B, inflammatory cells were observed 3 days post-administration, but decreased over time with almost no inflammation after 13 weeks. NiO C showed very mild lung effects at 3 days post-administration, and pulmonary inflammation disappeared 4 to 13 weeks post-administration. Neutrophil counts in BALF supported these trends in pulmonary inflammation for NiO A, B, C, and D (Table 7).

**Table 7:** Neutrophil counts in bronchoalveolar lavage fluid after intratracheal administration to rats

	Neutrophil counts in BALF [cells/μL]		
	Day 3	Week 4	Week 13
NiO A (6 mg/kg BW)	374 ± 125	446 ± 113	767 ± 465
NiO B (6 mg/kg BW)	534 ± 186	94.2 ± 60.8	0.363 ± 0.725
NiO C (6 mg/kg BW)	10.1 ± 4.44	4.10 ± 2.89	4.17 ± 6.60
NiO D (6 mg/kg BW)	391 ± 171	693 ± 379	1240 ± 472
Control	0.55 ± 0.88	0.33 ± 0.35	0.61 ± 0.73

## Discussion

In the dissolution test, only NiO B dissolved rapidly in artificial lysosomal fluid and more slowly in water, saline, and artificial interstitium fluid; this finding suggested that dissolution may occur in macrophage lysosomes following phagocytosis, and would occur more rarely outside macrophages. Pulmonary clearance rates were much faster in rats treated with NiO B than in those treated with NiO A, C, or D. The clearance rates of NiO B were much faster than previously reported fast clearance rate constants for insoluble particles [19, 20]. This suggested that the clearance pathway of NiO B was different from the clearance pathways of other insoluble particles. For NiO B, the relative percentage of the Ni found in BALF to total lung burden correlated inversely with the dose and increased over time, whereas those values were stable for the other three NiO nanoparticles. NiO B was excreted in the urine within 24 h of administration to a greater extent than the other NiO particles tested. In addition, the Ni ion/total Ni in BALF fraction for NiO B was higher than the corresponding values for NiO A, C, and D. These data suggest that the Ni ions dissolved from NiO B translocated from the lungs to the blood and kidneys, and were excreted in the urine as excretion of intravenously administered Ni ions has been shown to be rapid [21].

The pulmonary half-life (= ln2/*k*_Lung_) of NiO B was calculated as 4.4–4.5 days, and 310–410, 59–170, and 82–110 days for NiO A, C, and D, respectively. The half-life of NiO A, C, and D might be found to be longer if the observation period was set to be longer than that in the present study. This is because the effects of fast clearance decrease comparing to slow clearance over time. Previously, the pulmonary half-life of micron-sized, soluble NiSO_4_ particles was determined to be 1.6 h, while that of micron-sized, insoluble NiO and Ni_3_S_2_ particles was 92 and 90 days, respectively [22]. Differences observed between NiSO_4_ and NiO B might be attributable to the high solubility of NiSO_4_ in water, whereas NiO B is highly soluble in lysosomal fluid but only slightly soluble in water, saline, and interstitium fluid. The pulmonary half-life of six types of insoluble TiO_2_ particles has been reported as 34–44 days and 52–94 days for <2 mg/kg and 6 mg/kg, respectively, while that of Al(OH)_3_-coated TiO_2_ particles was 64, 141, and 907 days for 0.67, 2, and 6 mg/kg, respectively [2]. In addition, the slow clearance of NiO C and Al(OH)_3_-coated TiO_2_ particles may be explained by the toxicity of slightly dissolvable Ni or Al ions.

Particles in the alveolar region can be cleared by the following two routes: Route 1: Lung → phagocytosis by macrophage → macrophage transfer to the end of bronchi → tracheal ciliary motility after phagocytosis; route 2: Lung → phagocytosis by macrophage → phagosome-lysosome fusion in macrophage → dissolution in macrophage. NiO uptake via the alveoli into the pulmonary circulation was not assessed in the present study because intravenously administered insoluble TiO_2_ nanoparticles have been reported to be trapped in the liver [20], and the NiO burden in blood and liver was not high in the present study. Routes 1 and 2 operate in parallel, and events within each route occur in tandem. Therefore, *k*_*Lung*_ (/h) can be expressed using the phagocytosis rate constant, *k*_*phar*_ (/h), the macrophage migration to the end of bronchi rate constant, *k*_*mig*_ (/h), the phagosome-lysosome fusion rate constant, *k*_*fusion*_ (/h), and the dissolution rate constant in lysosomes, *k*_*dis*_ (/h), using the following equation:

4 $$ \frac{1}{k_{Lung}}=\frac{1}{k_{phar}}+\frac{1}{\frac{1}{\frac{1}{k_{dis}}+\frac{1}{k_{fusion}}}+{k}_{mig}} $$

**Fig. 7:**
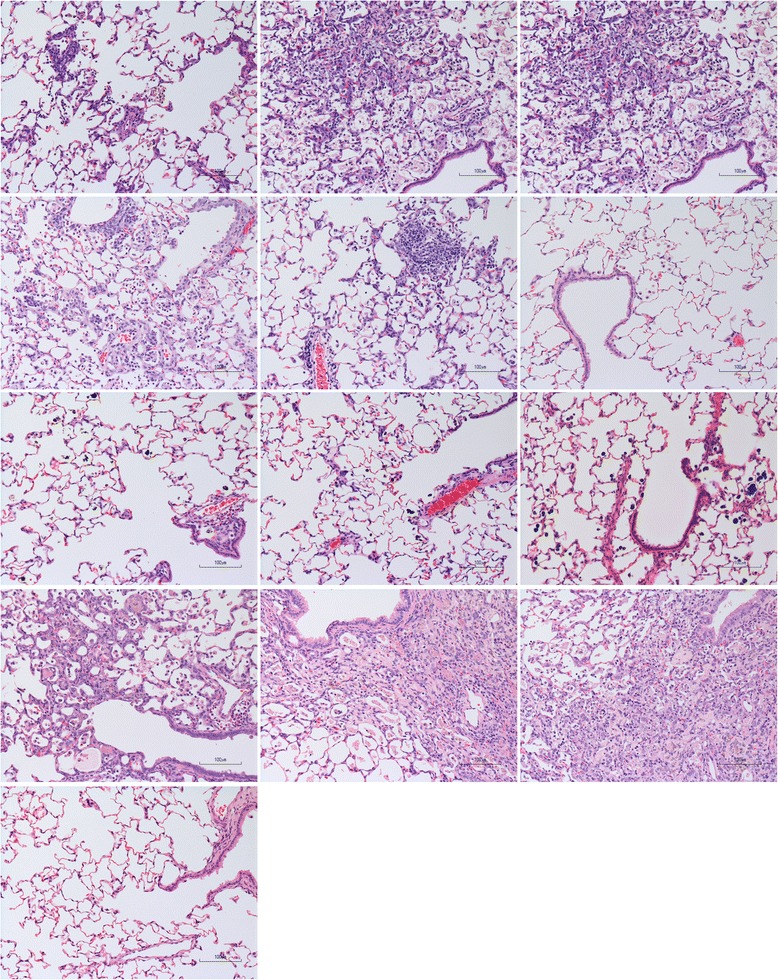
Estimated macrophage migration to end of the bronchi rate constant and phagosome-lysosome fusion rate constant. The estimate was calculated for 0.01, 0.05, and 0.1 /h dissolution rate constants in the lysosome

In the present study, as NiO nanoparticles were not observed outside macrophages in the microscopic observation on day 3, *k*_*phar*_ was estimated to be approximately 0.05/h. Therefore, assuming that *k*_*phar*_ is 0.01, 0.05, or 0.1/h (52, 98, and >99% of particles are estimated to be ingested by macrophages 3 days after exposure), *k*_*fusion*_ and *k*_*mig*_ were estimated using the determined values of *k*_*Lung*_ and *k*_*dis*_. Since *k*_*mig*_ was estimated to be constant with 1 × 10^−1^–1 × 10^−5^/h of *k*_*fusion*_ (Fig. 7) for NiO C only, the migration of macrophages after phagocytosis was suggested to be the rate-determining step (rate constant: 1 × 10^−4^–5 × 10^−4^/h). In other words, fusion and dissolution (Route 2) do not contribute to the pulmonary clearance of NiO C because of its low solubility in lysosomal fluid. This suggestion is in accordance with the dose-dependent overload observed for NiO C (only) in the animal experiment; this overload may reflect an inhibition of macrophage migration. For NiO A, B, and D, *k*_*fusion*_ or *k*_*mig*_ were estimated to increase in parallel with increasing solubility in lysosomal fluid (B > D > A) (Fig. 7). However, since overload was not observed in the animal experiment, particles located within macrophages did not affect *k*_*mig*_. Therefore, increases in *k*_*mig*_ with solubility cannot be explained, whereas increases in *k*_*fusion*_ with solubility appear to be reasonable, indicating that phagosome-lysosome fusion represents the rate-determining step. The *k*_*fusion*_ values were estimated to be 1 × 10^−4^, 1 × 10^−2^, and 2 × 10^−4^ for any concentration of NiO A, B, and D, respectively. Inhibition and/or promotion of phagosome-lysosome fusion depends on the proteins around the particles [23–25]. The adsorbed proteins on the particle can differ depending on the particle solubility.

For NiO B, the dissolution rate constant in lysosomal fluid (0.18/h) was much higher than that in interstitium solution (3.6 × 10^−3^/h), suggesting that NiO B dissolved in macrophages immediately following fusion. This is consistent with the findings that extracellular ion fractions in BALF were much higher for NiO B than for NiO A, C, or D. The elevated Ni concentrations in blood, kidney, and urine of animals treated with NiO B, compared with those receiving NiO A, C, or D, suggested that dissolved Ni was cleared via blood to the kidney and excreted in urine. The fairly low blood Ni level (< 1% 6 h after administration) indicated that this process occurred rapidly.

NiO translocation to thoracic lymph nodes increased in a time- and dose-dependent manner for NiO A, C, and D; this observation has also been reported for other insoluble particles (TiO_2_) [2, 26–28], but not for NiO B. These data suggest that dissolved Ni ions are distributed in the blood, but are not transferred to the lymph or immediately cleared from the lymph nodes.

The translocation of particles to lymph nodes indicates an overload due to damaged epithelial cells, as previously described [29]; particles that are cleared more slowly are transferred to the lymph nodes. In the present study, however, translocation to the lymph nodes was faster for NiO D than for NiO A, although pulmonary clearance rates were faster for NiO D than for NiO A. These phenomena are likely attributable to the greater solubility of NiO D in lysosomal fluid. Since Ni ions were not transferred to the lymph or immediately cleared from the lymph nodes, a possible mechanism for translocation to lymph nodes includes damage to epithelial cells, attributable to the Ni ion from NiO A and D, as well as from overloaded particles dissolving in the lysosome.

Particles that are soluble outside macrophages (e.g. NiSO_4_) have been shown to induce greater acute pulmonary inflammation than Ni compounds with lower solubility (e.g. Ni_3_S_2_, NiO, and Ni(OH)_2_) [13]. Ag ions are rapidly and directly toxic to organs, while nanoparticles induce toxicity more slowly, after dissolution in the lysosome [30]. Nanoparticles that are located within the macrophage and are rapidly soluble, such as NiO B, may induce acute pulmonary inflammation if the ion is toxic. Soluble nanoparticles located outside the macrophage can induce more severe inflammation than those within macrophages because the ion concentrations are much higher for the nanoparticles located outside the macrophage. However, this acute inflammation would decrease rapidly over time, because the dissolved Ni ions are rapidly cleared from the lungs [7, 8, 31]. In contrast, moderately soluble nanoparticles located within the macrophage, such as NiO A and D, may induce persistent pulmonary inflammation because of their longer retention and continual release of Ni ions in the lysosome. NiO particles that showed low solubility accumulated and induced chronic pulmonary effects in rats and mice, while water-soluble NiSO_4_ did not produce these effects [8]. In addition, nanoparticles such as NiO D, which dissolve slowly within macrophages induced only mild acute and persistent pulmonary inflammation. These results suggest that dissolution tests in biological fluids and ion toxicity data can provide valuable information on the acute and chronic toxicity of nanoparticles. However, nanoparticles with moderate solubility and highly toxic ions (such as NiO A and D) require animal testing using a relevant exposure route such as intratracheal administration to obtain toxicity and toxicokinetic data.

In the present study, we conducted intratracheal administration of nanomaterials on rats for the evaluation of inhalation exposure. There are some differences between rats and humans and between inhalation exposure and intratracheal administration. The fast clearance rate was not different between humans and rats, while the slow clearance rate was 2 or 3 times higher for rats than for humans [19] and the fast clearance rate was 1-order higher than the slow clearance rate [19]. Therefore, at least the magnitude relationship of the clearance rate did not switch under overload-level doses. In a previous study, there were no significant differences in multi-wall carbon nanotube retention between intratracheal administration and inhalation [32] and the treatment groups had the same ranking whether measured after intratracheal inhalation or after instillation of tracer particles [33]. Therefore, we can compare the clearance rate constants between different nanoparticles using intratracheal instillation test on rats.

## Conclusion

The present study measured the dissolution of nanoparticles in six different solutions, including artificial lysosomal fluid and artificial interstitium fluid. The tissue distribution and clearance of four types of NiO nanoparticles with different characteristics were determined 3, 28, and 91 days after intratracheal administration in rats. In particular, NiO nanoparticles that dissolve rapidly in artificial lysosomal fluid can be easily cleared from the lungs. With the exception of submicron NiO particles, pulmonary clearance overload was not observed, suggesting that the clearance mechanisms are not associated with macrophage migration to the end of bronchi, but involve dissolution in macrophage lysosomes.

## Additional files

Additional file 1: Contents of artificial lung interstitium fluid and artificial lysosomal fluid. The reagents were dissolved in pure water one by one beginning at the top (DOCX 20 kb)

Additional file 2: Recovery efficiencies from Ni-spiked samples (DOCX 18 kb)

Additional file 3: NiO burdens per initial body weight at the time of administration. Values for (A) lung, (B) bronchoalveolar lavage fluid (BALF), (C) trachea, and (D) lymph nodes are shown (DOCX 33 kb)

Additional file 4: NiO burdens per organ tissue weight. Values for (A) lung, (B) bronchoalveolar lavage fluid (BALF), (C) trachea, and (D) lymph nodes are shown (DOCX 33 kb)

Additional file 5: Percentage of Ni in each lymph node out of the total Ni in all lymph nodes (DOCX 19 kb)

## Abbreviations

ANOVA: Analysis of variance
BALF: Bronchoalveolar lavage fluid
BW: Body weight
ICP-MS: Inductively coupled plasma-mass spectrometry
NiO: Nickel oxide
