# Supplementary material for: Kinetics and dissolution of intratracheally administered nickel oxide nanomaterials in rats
Source: Part Fibre Toxicol. 2017 Nov 28;14:48. doi: 10.1186/s12989-017-0229-x (PMC5706298; doi:10.1186/s12989-017-0229-x)
Supplement: Supplementary file 1 — Contents of artificial lung interstitium fluid and artificial lysosomal fluid. The reagents were dissolved in pure water one by one beginning at the top (DOCX 20 kb) [file 12989_2017_229_MOESM1_ESM.docx]

**Additional file 1. Contents of artificial lung interstitium fluid and artificial lysosomal fluid.** The reagents were dissolved in pure water one by one beginning at the top.
