# Supplementary material for: Kinetics and dissolution of intratracheally administered nickel oxide nanomaterials in rats
Source: Part Fibre Toxicol. 2017 Nov 28;14:48. doi: 10.1186/s12989-017-0229-x (PMC5706298; doi:10.1186/s12989-017-0229-x)
Supplement: Supplementary file 2 — Recovery efficiencies from Ni-spiked samples (DOCX 18 kb) [file 12989_2017_229_MOESM2_ESM.docx]

**Additional file 2: Recovery efficiencies from Ni-spiked samples.**

* BALF: bronchoalveolar lavage fluid

** 5 ng/mL of Ni standard solution were added to an actual organ samples per every 10 to 20 samples and the measured value were corrected by the recovery efficiency.
