# Supplementary material for: Kinetics and dissolution of intratracheally administered nickel oxide nanomaterials in rats
Source: Part Fibre Toxicol. 2017 Nov 28;14:48. doi: 10.1186/s12989-017-0229-x (PMC5706298; doi:10.1186/s12989-017-0229-x)
Supplement: Supplementary file 3 — NiO burdens per initial body weight at the time of administration. Values for (A) lung, (B) bronchoalveolar lavage fluid (BALF), (C) trachea, and (D) lymph nodes are shown (DOCX 33 kb) [file 12989_2017_229_MOESM3_ESM.docx]

**Additional file 3: NiO burdens per initial body weight at the time of administration. Values for (A) lung, (B) bronchoalveolar lavage fluid (BALF), (C) trachea, and (D) lymph nodes are shown.**

**(A)Lung**

**(B) Bronchoalveolar lavage fluid (BALF)**

**(C) Trachea**

**(D) Lymph nodes**
