# Supplementary material for: Kinetics and dissolution of intratracheally administered nickel oxide nanomaterials in rats
Source: Part Fibre Toxicol. 2017 Nov 28;14:48. doi: 10.1186/s12989-017-0229-x (PMC5706298; doi:10.1186/s12989-017-0229-x)
Supplement: Supplementary file 4 — NiO burdens per organ tissue weight. Values for (A) lung, (B) bronchoalveolar lavage fluid (BALF), (C) trachea, and (D) lymph nodes are shown (DOCX 33 kb) [file 12989_2017_229_MOESM4_ESM.docx]

**Additional file 4. Additional file 4: NiO burdens per organ tissue weight. Values for (A) lung, (B) bronchoalveolar lavage fluid (BALF), (C) trachea, and (D) lymph nodes are shown.**

**(A) Lung**

**(B) Bronchoalveolar lavage fluid (BALF)**

**(C) Trachea**

**(D) Lymph nodes**
