# Supplementary material for: Kinetics and dissolution of intratracheally administered nickel oxide nanomaterials in rats
Source: Part Fibre Toxicol. 2017 Nov 28;14:48. doi: 10.1186/s12989-017-0229-x (PMC5706298; doi:10.1186/s12989-017-0229-x)
Supplement: Supplementary file 5 — Percentage of Ni in each lymph node out of the total Ni in all lymph nodes (DOCX 19 kb) [file 12989_2017_229_MOESM5_ESM.docx]

**Additional file 5. Percentage of Ni in each lymph node out of the total Ni in all lymph nodes.**
